# 

**Published:** 1893-12-02

**Authors:** 


### CONTENTS.

**The Royal Hospital for Incurables** - 129
**Inhalation of Oxygen for Therapeutical Purposes.** By Sir B. W. RICHARDSON, M.D., F.R.S. - 131
**The Medical Work of the Local Government Board.** VI.—Inoculation and Insusceptibility - 132
**Modern Biology.** IV.—Cell Division - 133
**The Field of Science** - 134
**Notes and News** - 136
**The Hospital Clinic**—
THE TREATMENT OF ACUTE MANIACAL DELIRIUM - 137
ROYAL ORTHOPÆDIC HOSPITAL.—Treatment of Contraction of the Fingers - 138
EDINBURGH ROYAL INFIRMARY—Recent Uses of Thyroid Preparations - 139
**Medical Protection and Defence** - 139
**Annotations.**—Public School Discipline—A Poor Man's Church—Schoolboy Students - 135
**The Institutional Workshop**—
HOSPITAL CONSTRUCTION—Hospital Convalescent Home, Parkwood, Swanley, Kent - 141
PRACTICAL DEPARTMENTS.—Ward Cupboards.—I. Medicine Cupboards - 142
HOSPITAL FESTIVALS AND MEETINGS—The Metropolitan Hospital Sunday Fund—The East London Hospital for Children—Royal Hospital for Incurables - 143
CONSTRUCTION NOTES - 144
**Scraps and Gleanings** - 144
**The Book World of Medicine and Science** - vii

### EXTRA SUPPLEMENT.—THE NURSING MIRROR.

**News from the Nursing World.**—Christmas Festivities—Our Christmas Competitions—Fair Pay—Thoroughly Trained—Her Luggage—Wanted, a Well Qualified Nurse—Queen's Nurse at Selkirk—An Eloquent Appeal—Training at Philadelphia—India—Influenza in Victoria—Short Items - lxxxi
**On the Nursing of Diseases of the Stomach.** IV.—Hæmatemesis - lxxxiii
**Women's Work.**—Female Medical Aid for the Women of India. By MRS. SCHARLIEB, M.D. III.—The Training Necessary and Procurable - lxxxiv
**Nursing in the United States.**—Training School for Nurses of the New York Hospital—The Nurses' Home - lxxxiv
**Nursing in Switzerland.** By Dr. CHARLES KRAFT - lxxxv
**Wants and Workers** - lxxxiii
**The New York City Training School for Nurses, Blackwell's Island** - lxxxv
**Nursing in Japan** - lxxxvi
**Cottage Hospitals in the West Country.**—Lyme Regis - lxxxvi
**Everybody's Opinion.**—Unique Testimonials—Legally Entitled to Her Certificate—Asylum v. Hospital Training - lxvxvii
**Medico-Psychological Association of Great Britain and Ireland.** Written Examination for Nursing Certificate, November, 1893 - lxxxvii
**In the Canaries.** By a PATIENT. Santa Cruz - lxxxviii
**Appointments** - lxxxviii
**The Book World for Women and Nurses** - lxxxix

